# A Fasciculus of Nine Lithographic Anatomical Drawings, from Preparations in the Museum of the Army Medical Department at Chatham

**Published:** 1825-07-01

**Authors:** 


*A Fasciculus, containing nine Lithographic Anatomical Drawings; from Preparations in the Museum of the Army Medical Department at Chatham.* Dedicated to his Royal Highness the Commander in Chief, &c. Folio, 1824.

THE attention of the army medical officers was particularly drawn to *post-mortem* inspections, in the year 1816, when the olive-branch had happily taken the place of the laurel, and when a period of profound peace was justly deemed peculiarly eligible for commencing a collection in morbid anatomy, and thus contributing to pathological knowledge. Through the patronage of His Royal Highness, the Duke of York, an apartment in the hospital buildings, at Chatham, was appropriated to this desirable purpose, and, since the period alluded to, the success of the undertaking has exceeded the most sanguine expectations. The museum already contains many drawings and paintings in oil, illustrative of diseased appearances, by Mr. Schetky, whose premature death the army and the profession at large have now to deplore. This most meritorious officer fell a sacrifice to the deleterious climate of Africa—to which vile place it is a great pity he was ever sent! To the deceased, (jointly with Mr. Millar) aided by the talents of Drs. Forbes, Skey, Davy, and others, the museum owes its present advanced and flourishing condition.

We are most happy to inform our brethren, especially of the naval department, that, through the exertions of the present medical commissioners of the navy, similar establishments are forming at Haslar and Plymouth Hospitals, where libraries are to be accumulated, and clinical lectures to be delivered to the young naval medical officers, by the physicians and surgeons of those institutions. This is as it ought to be—to have long been—but better late than never.

To return. It having been suggested that a selection from the most important subjects (in the Chatham Museum) represented by correct drawings, might be advantageously made, these plates have been executed and published with that view. They are illustrated by descriptions and by histories of the several cases, abstracted from the detailed clinical reports preserved in the library of the Military Hospital.

Having thus sketched out the objects and nature of the work, we shall now proceed to notice some of the cases which it contains.

1. The first plate is illustrative of an abscess of rather unusual site—namely, in front of the spine, extending from the 6th cervical to the 8th dorsal vertebra, containing 18 ounces of purulent matter. The aorta and œsophagus were thrown in front of this large sac—the bodies of the vertebræ were in a carious state, and the intervertebral substance in a great degree absorbed. The patient was only 23 years of age, but of a scrofulous constitution, and completely broken down by West India fever. A slight curvature was perceived in the dorsal region of the spinal column, and a tumefaction with obscure fluctuation, without external inflammation or pointing to the left of the spine. No change in this tumefaction afterwards took place.—The hectic symptoms bespoke internal suppuration, but the patient sunk under an attack of acute bronchitis. The *post mortem* appearances we have stated above. The plate is necessarily obscure, from the parts being in such a state of destruction.

2. The second plate contains two figures, coloured, and very beautifully representing inflammation of the theca of the spinal cord.

*Case* 1. J. O. a dragoon, received a contusion on the loins by a fall from his horse, producing paralysis of the lower extremities, bladder and rectum, in which state he was received into hospital, in February 1822. The effects of the constant dribbling of the urine were obviated as much and as long as possible, by various expedients; but the gangrenous excoriations invaded every fresh part exposed to pressure in the changes of position which took off the pressure from other parts. He sunk under these irritations in the spring of the succeeding year.

"On dissecting the inferior extremities, the cellular substance was found to be destroyed by gangrene in the form of slough, macerated in fetid pus, to a much greater extent than was indicated before death by the apparent extent of the ulcerated abrasions. The muscles of the thigh and ham were extensively stripped of their investing adipose membrane. In the left knee-joint the articulary heads of the bones were found denuded of their cartilages by ulceration. The cancellated bony texture was extremely soft, and the bony shell thin, soft, and yielding, so as almost to sink under the pressure of the saw. The fasciæ and ligamentous apparatus of the joint were involved in the same common destruction as the harder parts.

"The muscles of the paralytic limbs were every where pale, and in many places converted into a substance presenting the qualities of fat or suet. The fat was also very plentiful in those parts of the limbs which had not been attacked by the fetid suppurations, while in the upper part of the body its quantity was not unnatural. The nerves of the lower limbs were not diminished in size.

"On laying open the spinal canal, it was observed, generally, that the medulla spinalis was unusually soft; and at the situation of the first dorsal, and sixth and seventh cervical vertebræ, the theca was discovered to be, at least, thrice its natural thickness, and exhibiting marks of increased vascularity, whilst the medullary matter of the chord itself was found to be converted into a dusky, semi-transparent, and yellowish substance, somewhat resembling paste or jelly, both in consistence and colour.

"The other regions of the body and the viscera were found natural, after a minute inspection." 2.

*Case* 2. J. B. (ætat. 60) was received into hospital on the morning of September 6, 1820, with his right arm enormously swelled from the hand to the shoulder, and, in different parts, of a bluish colour, with small vesications. He was speechless, and no history of the symptoms could be obtained. His left arm was continually agitated by spasms—his head drawn to the left side,—and at intervals there were similar sudden retractions of the muscles of the back, resembling opisthotonos. He expired the same day. It was afterwards ascertained that he was ill only one entire day in barracks, previously to his entrance into hospital. Under such circumstances, the following *post mortem* appearances will excite the surprise of the reader.

"The vessels of the head, and the substance of the brain, were very turgid with blood. The ventricles did not contain any unusual quantity of fluid. The cerebellum had the same vascular appearance as the cerebrum. The vessels in the spinal canal were gorged with blood, and the theca of the medulla spinalis was very red; the inflammation, however, did not extend to the substance of the spinal marrow itself.

"The lungs were completely filled with blood; the lining membrane of the bronchiæ was reddish. There was also some effusion into the cavity of the pleura. The internal tunic of the heart and blood-vessels (particularly the inner coat of the arch of the aorta), was of a brilliant red; the valves were natural; the pericardium contained the usual quantity of fluid." 2.

The third plate contains four figures, all representing disease of the bones after syphilis, or what is termed pseudo-syphilis. Of these we can convey no idea in words, and must therefore refer to the plates themselves.

The fourth plate contains two figures—the first, a continuation of the subject of the preceding plate,—and the second, a representation of syphilitic, pseudo-syphilitic, or mercurial ulcers on the toes and soles of the feet.

5. The fifth plate contains four figures very ably executed, and also coloured. The first figure represents a dreadful case of ulceration of the nates. The patient was a journeyman tailor in civil life, in Edinburgh, who had had syphilis—two chancres on the penis, and two buboes, the chancres having appeared eight days after suspicious connexion. Mercury (by inunction) was used, to the effect of producing slight soreness of the mouth, but no salivation. This treatment was continued for two months, and the symptoms yielded. While the buboes were healing, and about six months after the commencement of the mercurial course, a spot of a bright red colour, neither painful nor elevated, appeared on the left side of the perineum, and speedily ulcerated. He again used mercury, in the form of pills. About a month after the commencement of this second course, a small tumour, the size of a bean, appeared on the right hip, which also broke and ulcerated, the ulceration spreading into the previous sore in perineo. The pills only produced a disagreeable taste in the mouth.

"The elongated ulcer was attended with burning pain; worse at night; it extended under the use of these pills; he was then ordered to take bark and to intermit the pills. The bark was used for three months, and appeared to aggravate the disease.

"He then abandoned all internal medicine, and used hot stimulating dressings.

"The sore spread over the ischium of the right side, and healed at one point in its centre. He was sent into the country. No progress being made in the cure within a month afterwards, mercury was used, by inunction, morning and evening, for a month, but produced no affection of the mouth, except a slight soreness. The ulcer spread slowly and steadily; healing in the centre. Its progress continued uninterrupted after the cessation of the mercury. Once more he used mercury, which produced salivation. The ulcer still spread, and encircled the anus. He now used nitric acid internally, and nitrate of silver in solution, as an external application, alternately with a mixture of calomel and lime water.

"At this period the painting was taken. The ulceration continued to enlarge towards the circumference, and heal in the centre.

"The last time I saw him (in Musselburgh, in 1819,) it had ascended as high as the middle of the sacrum, and descended more than half down the thighs. He was then hectic, and appeared to be dying."

The other figures represent disease of the cranium in worn-out soldiers returned from the East and West Indies. One of them related that he never had had syphilis—but as for the assertions of these people, we should not place the slightest reliance on them. It would be a rare thing indeed to see a soldier reside 12 or 15 years in the East or West Indies, without contracting any syphilitic taint.

6. The sixth plate, which is coloured, represents an eruption covering the whole body, but more particularly the head and neck. It appeared four or five days previous to the patient's admission. Its nature was pustular, with a dark-coloured areola. These pustules became deep-set crusts, on the removal of which sores were found. He had also extensive ulcers in the fauces, which gave him much pain, and greatly impeded his swallowing. He had been with his regiment in India for ten years, during which he had undergone a course of mercury for some of the diseases of the climate; but never, as he said, for syphilitic complaint. He sunk, emaciated, under hectic. Extensive ulceration was found in the nasal membrane—and also on each side of the tongue. The epiglottis was nearly destroyed—the uvula completely so. The lungs were hepatized.

7. The seventh plate contains four figures, three of them illustrative of disease of bone after syphilis and mercury. The 4th figure is meant to represent fracture of the neck of the thigh bone united. The man was 31 years of age and died of phthisis. About four years previously he had a fall from his horse, in America, and fractured both of the inferior extremities. The fracture of the right thigh being compound, amputation was performed. The left was found with a fracture of the middle and neck of the femur, "in which bony union had completely taken place." Considering the age of the patient, we dare not say that the appearance of the neck of the thighbone is not proof of fracture, though we certainly have doubts on the subject, having attentively examined the bone itself, from which the drawing is made. But, at all events, there can be no doubt that the fracture (if it be one) is external to the capsular ligament, at least in part, and, consequently, does not bear on the controversy between Sir Astley Cooper and Mr. Earle, respecting the union or non-union of the bone within the capsule. We have had the opinions of some of the ablest anatomists in this metropolis on the bone in question, and the result has been unfavourable to the statements, or rather opinions, broached in this Fasciculus. We would advise the plate to be cancelled, or the opinions to be modified.

9. The ninth plate (the 8th is a continuation of the 7th) exhibits specimens of diseased intestines and lungs, after dysentery and phthisis; but it is so difficult to represent morbid parts of this kind by lithographic plates, that they fail to convey any clear idea of the morbid structures.

We have now given a brief account of the present Fasciculus, and have only to wish success to the undertaking.

